# Examining the effects of psychological reactance on COVID-19 vaccine acceptance: Comparison of two countries

**DOI:** 10.7189/jogh.12-05057

**Published:** 2022-12-21

**Authors:** Fahimeh Hateftabar, Heidi J Larson, Vahideh Hateftabar

**Affiliations:** 1 Management Department, University of Paris 1 Pantheon-Sorbonne, Paris, France; 2 Anthropology, Risk and Decision Science, London School of Hygiene & Tropical Medicine, London, UK; 3 Vaccine Confidence Project, London School of Hygiene & Tropical Medicine, London, UK; 4 Global Immunisation Communication, UNICEF, New York, USA; 5 Department of Health Metrics Sciences, University of Washington, Seattle, Washington, USA; 6 University of Antwerp, Antwerp, Belgium; 7 Department of ENT, Faculty of Medicine, The University of Tabriz, Tabriz, Iran

## Abstract

**Background:**

Despite the public acceptance of COVID-19 vaccines being necessary to achieve ample immunization rates and, in turn, put an end to the global pandemic, vaccine hesitancy and refusal are on the rise. To detect and address the concerns of those who are hesitant, it is critical to identify all potential factors behind vaccine decision-making in order to devise strategies to enhance vaccine acceptance and uptake.

**Methods:**

We retrieved a total of 742 and 685 completed questionnaires from Iran and France, respectively; after initial cleaning and data screening, the number of usable questionnaires dropped to 714 and 664. We evaluate the distinct vaccination and lockdown restrictions in Iran and France and used multi-group analysis to evaluate structural path models of French and Iranian people, revealing a significant difference between the two groups in vaccination-related decisions. This empirical study is one of the first to employ the measurement invariance was evaluated using the measurement invariance for composite (MICOM) approach in testing partial least squares-structural equation modelling (PLS-SEM) measurement invariance and one of the first to use both Henseler’s MGA and Henseler’s permutation method to perform multi-group analysis (MGA).

**Results:**

MGA revealed significant differences in the effects of influential factors on vaccine acceptance across France and Iran. In other words, many determining factors are likely to be context-dependent. The study revealed that reactance (due to restrictions and perceived scarcity) and financial strain significantly influenced COVID-19 vaccine acceptance and uptake among Iranians; however, among the French, confidence, and convenience were far more influential.

**Conclusions:**

These cross-cultural differences point to the importance of conducting additional research in this area that directly compares various effects across countries. Each country’s public health authorities and policymakers could use these insights to develop more targeted strategies and, in turn, boost vaccination rates among the public.

The ongoing global pandemic, triggered by SARS-CoV-2, has had far-reaching health, social, and economic consequences, including limitations on our daily movements, connections, and activities. By August 27, 2021, the virus had reached all nations and territories, infected about 216 million individuals, and killed over 4 million people globally. At the onset of this crisis, most nations employed physical distancing and other strategies to control the spread of COVID-19 and, in turn, its death toll [1]. The intention was for these policies to be maintained until herd immunity was achieved, meaning SARS-CoV-2 could no longer be transmitted. Herd immunity, stemming from high vaccination coverage, has played an essential role in mitigating and even sometimes eradicating the endemic transmission of various illnesses, benefiting not only vaccinated people but society as a whole. There are two challenges facing the attainment of herd immunity: making vaccination accessible and convincing people to get vaccinated [2]. In other words, immunization effectiveness is achieved by high levels of both coverage and acceptance [2]. High rates of vaccine efficacy and safety are predicted to be associated with lower levels of hesitation, but COVID-19 vaccine hesitancy and refusal are increasing throughout the world regardless [3]. This dynamic may ultimately squander the substantial effort that was devoted to the development of the COVID-19 vaccines in their final stages [4,5]. Vaccine refusal raises the risk of disease for unvaccinated individuals and their entire community. Thus, identifying, analysing, and resolving the issues of vaccine hesitancy and refusal are crucial steps to boost vaccine uptake and, in turn, put an end to the pandemic.

Public acceptance of vaccines has emerged as a key topic in medical and social science research [5-8], in large part due to growing evidence of COVID-19 vaccination refusal [3,5]. A thorough examination of the literature on willingness to receive the COVID-19 vaccination reveals several gaps. First, a vast majority of studies on this issue have been done in high-income nations [2,9-13] or, to a lesser degree, middle-income countries [14-16]; little is known about vaccination willingness in low-income nations [17]. This disparity has prompted calls for more studies in low-income countries to analyse their antecedents of vaccine acceptance/refusal [17]. The COVID-19 pandemic has raised scholarly awareness of the need to understand complex political, social, and behavioural factors that influence public acceptance of vaccination [7]. Prior research indicates that different contexts generally host different public behaviours regarding vaccination, the underlying causes of which vary considerably by country. Thus, identifying the importance of different components across contexts would enable targeted and tailored vaccine-uptake interventions [18]. One of the main differences between high-income countries and low-income countries (in most cases) relates to their strategies regarding state financial support during lockdowns. Of course, over the course of the COVID-19 pandemic, all countries, particularly low-income countries, faced serious challenges managing the livelihood of their people. In addition to the health crisis, people faced an unprecedented economic crisis brought about by the emergence of the disease. Previous studies suggest that, when financially strained, people modify their behaviours and perceptions [19,20]. During lockdowns, most of the high-income countries provided financial support to their citizens to mitigate their financial difficulties. As financial strain has been shown to influence behaviours and perceptions, the restriction of vaccine-willingness literature to high- and middle-income countries neglects a critical variable and, in turn, may lead to the implementation of suboptimal or inappropriate strategies in contexts with more limited means to providing financial support to their citizens.

In addition, as vaccine willingness is multifaceted, complex, and driven by a variety of emotional, social, cultural, political, and religious variables [7,21], several studies have sought to identify further determinants of COVID-19 vaccine willingness [7,10,11,15,22]. Despite the well-established fact that scarcity and prohibition (which both lead to psychological reactance) influence individuals’ behavioural intentions and decisions [23-28], no study has yet to explore their effect on individuals’ intentions to accept, hesitate about, or refuse COVID-19 vaccination. This study suggests that these two factors, which both lead to psychological reactance, are among the factors that influence individuals’ vaccination-related behaviours and decisions. Our study is grounded in [29] psychological reactance theory (PRT), which states that individuals respond to limitations imposed on them by raising the value that they assign to the limited item or behaviour, and [30] commodity theory, which asserts that the attractiveness of goods increases alongside their scarcity. As a result, it is reasonable to believe that these two factors can impact people’s perceptions and behaviours regarding vaccination. Our original conceptual framework introduces and identifies a new direction for research in the field of vaccine willingness.

We test our hypothesized relationship in Iran and France. Our choice of Iran as the Lower-middle-income case study stems from calls to emphasize diverse contexts motivated by [17] who called for greater emphasis on various contexts. Additionally, it pairs well with our hypothesis. The Iranian government itself is struggling with a fiscal deficit, international sanctions, and a substantial decline in the global price of oil. These issues have made it difficult for the government to provide its people with financial support and protection during the pandemic. Unlike in high-income countries, in middle- and low-income countries like Iran, financial resources are extremely limited [19]. Many people lost their incomes due to the pandemic, and their governments were unable to compensate them. The official count of employed people in Iran dropped by 2.5 million in 2020 compared to 2019. According to official statistics, about 700 000 people registered to receive unemployment insurance in 2020 – far lower than the number of people who became unemployed due to the pandemic. The Iranian Parliament Research Center reports that more than 60% of working people are not covered by any type of insurance, meaning they cannot legally demand supportive accommodations. The pandemic forced Iran, a country facing financial crisis long before COVID-19, to struggle to cope with its dreadful circumstances. In countries accounting for approximately 75% of the global economy, governments proposed relief packages equal to 5% of the global GDP. In Iran, the government only managed a stimulus equal to 0.2% of its GDP.

Moreover, Iranian politicians conflate foreign policies and public health and prevent imports of British and American vaccines. As a result, Iranian vaccination (using Chinese and Russian vaccines) has gone very slowly, with only 4% of the population (less than 4 million people) having received their first dose through June 3, 2021. Other COVID-19 vaccines (eg, Pfizer-Biontech and Astra Zeneca) are prohibited.

For our high-income comparison, we use France. The French government prepared expansive vaccination plans. The country wields enough vaccines for the entire population, and the government gives them to people free of charge. Through June 3, 2021, 41% of the population (27 million) had received their first dose. As with most high-income countries, the French government compensated its people during lockdowns and implemented various forms of financial support for businesses.

Importantly, there is a significant difference between Iran and France in terms of COVID-19 vaccine willingness. Vaccine hesitancy in France has been identified as being one of the most vaccine-hesitant countries in the world [31-33]. Fifty-seven percent of French people hesitate to get vaccinated for COVID-19 – but this number is far lower among Iranians at about 6%. Reports indicate that, within the span of a single month (June 2021), more than 14 000 Iranians – frustrated with their government’s chaotic vaccine rollout and desperate for protection after enduring wave after wave of the coronavirus - travelled by air or land to neighbouring Armenia to be vaccinated against COVID-19. Iranians were willing to deal with the cost and stress of travel to get a vaccine. Iranians also demonstrate a high willingness to get vaccinated in Iran, with many Iranians waiting in queues of hundreds of meters long to be vaccinated.

This research represents an essential contribution to the literature on the relationship between COVID-19 vaccine willingness and perceived scarcity, prohibition, and perceived financial crisis in cross-cultural contexts. Such comparisons between two various contexts is crucial to uncovering the determinants of vaccine willingness among different peoples in different contexts and, in turn, developing more targeted public health messaging and reducing public health disparities between the developed and developing worlds.

## Vaccine acceptance models

Vaccine acceptance and hesitancy are the most important variables behind vaccination coverage. Vaccine hesitancy, as it is commonly known, is a global problem [31-33]; as such, national and international health agencies have collaborated with academia to study its origins and mitigate its implications. Hesitancy defines a continuum from acceptance to complete opposition [21]. Over the last decade, several academics have raised alarms over a global decline in public trust in vaccination and a global increase in vaccine hesitancy [33]. Vaccine-hesitant individuals have been characterized as a diverse group at the centre of a spectrum from vax-accepters to anti-vaxxers. These “hesitant” persons may oppose some vaccinations while agreeing to others, postpone immunizations, or accept vaccinations while being uncertain about the choice [34]. The World Health Organization (WHO) requests that nations monitor and report on vaccine hesitancy to continually track trends and discover vaccination concerns early [35]. The first global systematic effort was made by a group in the WHO Advisory Committee (Strategic Advisory Group of Experts on Immunization, SAGE), defining vaccine hesitancy as delay in accepting or refusing vaccinations notwithstanding the accessibility of vaccine-related services. Vaccine hesitancy is complicated and context-dependent; it varies by place, time, and virus against which the vaccination inoculates [7,21]. This study considers vaccine willingness to be influenced by many variables, including confidence (trust in vaccines or providers); people who lack confidence hold negative views of vaccination and are less inclined to get vaccinated. Complacency arises when the risks related to vaccine-preventable disease and vaccine values have limited perceptibility. In other words, complacent people do not perceive themselves to be at risk of infectious disease; therefore, they do not feel incentivized to change their preventative behaviours. Other important factors are convenience and constraints (physical accessibility, affordability, and perceptibility). Furthermore, at least in certain nations, these factors seem to vary little across sociodemographic groups [36]. Such findings led to the establishment of a Vaccine Confidence Index (VCI) survey tool in 2016 to assess individual attitudes toward vaccine safety, necessity, effectiveness, and religious compatibility. The VCI focuses primarily on measuring confidence across multiple countries through a minimal approach, thus enabling its straightforward integration into existing global surveys [33]. The 3-C model – that of confidence, complacency, and convenience – has been applied most frequently by researchers to study the formation of people’s vaccination behaviours. However, a recent body of literature has integrated new components into the 3-C paradigm. For example, [37] added another C-calculation to incorporate individual engagement in extensive research, forming a 4-C model. In 2018, [38] added collective responsibility as another psychological antecedent, resulting in a 5-C model. These models and their extended variants offer insight into why certain individuals get vaccinated while others refuse to do so, meaning they highlight potential barriers to vaccination [38].

Numerous psychological concepts have been explored in relation to vaccine willingness. For example, researchers have found that altruistic beliefs [11,39], the personality traits neuroticism and conscientiousness [40,41], health locus of control [11,42], subjective norms [43,44], personality trait agreeableness [11], cognitive reflection [45], and conspiracy perceptions [11,46], in some form, influence vaccine willingness. As previously stated, the COVID-19 pandemic has raised global awareness of the need to study the many diverse variables driving public vaccination acceptability. The growing emphasis by researchers on the complexity behind behavioural responses calls for an investigation into the roles of perceived scarcity, prohibition (which both trigger psychological reactance), and financial crisis. Brehm coined the PRT in 1996, asserting that people frequently act in opposition to imposed restrictions or pressure. He argued that reactance affected subjective attractiveness. This idea was explored further [47,48], leading to the PRT gaining significant attention from researchers of individual behaviours and intentions. Several scholars concluded that individuals react against imposed restrictions by raising the value that they assign to the limited item or behaviour [23,24,29,49]. Researchers from various disciplines contend that, if people’s freedoms are endangered, they are inclined to engage in menacing behaviour [23,29,49]. Once people experience psychological reactance, they are often driven to re-establish their independence by enhancing the attraction of the restricted item or behaviour. For example, prohibiting consumer products may improve their attractiveness to customers [48]. In addition, according to commodity theory [30], the desirability of products grows alongside scarcity, as scarcity makes a commodity look more appealing. When items, knowledge, or health conditions are scarce, the freedom to obtain them is endangered [50,51], triggering a reaction. Thus, desired but limited vaccinations may cause reactance, influencing behaviour to compensate for the constrained freedom. As a result, the incentive to regain the constrained freedom grows, resulting in efforts to acquire access to the rare resource [50]; simultaneously, the subjective worth of the good rises. Thus, both conditions in Iran (the prohibition of British and American vaccines and the very low speed of vaccination) lead to reactance behaviour. Psychological reactance is often considered to be one of the most prominent constructs in human behaviour and a strong determinant of decision-making [23,25-28,49]. We aim to investigate the influence of state financial assistance during pandemic lockdowns. While many governments compensated their people, certain countries like Iran [19] were unable to provide financial assistance to those who lost their earnings as a result of the pandemic. In other words, due to the degree to which the pandemic lockdowns endangered Iranian citizens’ financial circumstances, Iranians are less likely to ignore the cause of the problem (they show greater predisposition to get vaccinated).

In light of this assessment, this study employs the 3-C model, expanding on it by integrating three less-studied constructs, state financial support, perceived scarcity, and restriction on freedom, to analyse individuals’ behaviours toward COVID-19 vaccination uptake. We added these variables because all have been recognized in the literature as effective predictors of individual behaviour and decision-making. Additionally, most of the lower-middle-income countries struggled with these issues (vaccine scarcity and limited state financial support); it is not logical to neglect the impacts of these variables on individuals’ attitudes toward vaccination.

There is a dearth of research on COVID-19 vaccination behaviour among Iranians. Authorities merely report the high rate of vaccine acceptance without understanding its underlying reasons. This information is urgently needed to understand the current vaccination situation and facilitate more efficient vaccination. Furthermore, comparative studies on COVID-19 vaccine adoption in low- and high-income countries are rare. Such research would enable authorities to align public health messaging more closely with the specific psychological dispositions of vaccine-hesitant people.

## METHODS

This study employed a quantitative approach. We developed an online, self-administered questionnaire based on extant literature using a five-point Likert scale (1: “strongly disagree”; 5: “strongly agree). The data for this research were collected from April to June 2021. Translation/back-translation techniques were used to translate questionnaires for non-English speaking samples into their native language; issues regarding understanding and translatability were raised and addressed throughout the process. Additionally, we conducted a pilot test with 30 respondents and checked the reliability using Cronbach’s alpha. Results revealed a Cronbach’s alpha greater than 0.8 for all constructs, indicating acceptable reliability. We retrieved a total of 742 and 685 completed questionnaires from Iran and France, respectively; after initial cleaning and data screening, the number of usable questionnaires dropped to 714 and 664 (Table “S1” in the **Online Supplementary Document**).

Previous research suggests that the sample threshold for partial least squares-structural equation modelling (PLS-SEM) can be as low as 100. Given this standard from [52], our sample sizes are more than sufficient for reliable data analysis. However, the more limited minimum sample size suggested according to statistical power may also be used. We used G*Power to compute the sample size according to statistical power, which indicated that we required 136 samples for model testing with statistical power of 0.95. Once again, our sample sizes are more than sufficient. Given our adoption of a self-administered survey, we also tested for common method bias (CMB), in line with the recommendations of [53]; to ensure that we would gather honest and reliable responses, we conducted a voluntary and anonymous survey. Moreover, we employed Harman’s single-factor test with seven constructs and their items to detect CMB. We conducted exploratory factor analysis by loading all measurement items through unrotated principal components factor analysis. The results revealed that 32.61% and 26.4% – for Iran and France, respectively – of the variance was explained by the first factor, less than the recommended level of 50% [53]. Furthermore, we employed the unmeasured method factor technique to test CMB. For Iran, the average variance was 62%, while the average method-based variance was 1.4% (44:1). For France, the average variance was 74%, while the average method-based variance was 1.5% (49:1). Thus, CMB does not affect this investigation.

As indicated in **Table 1**, the mean values for COVID-19 vaccine acceptance were higher for the Iranian respondents than the French respondents. This indicates that French citizens are more reluctant to get vaccinated than the Iranian. Moreover, convenience had the lowest mean value (mean (m) = 2.15) for Iranians, likely due to their country’s deprivation and shortage of vaccines. The results for Iran demonstrated the highest value for restrictions (m = 4.32), followed by financial strain during the pandemic lockdowns (m = 3.87), perceived scarcity (m = 3.77), confidence (m = 3.1), complacency (m = 2.62), and, lastly, convenience (m = 2.15). The results for France demonstrated the highest value for confidence (m = 3.9), followed by convenience (m = 3.63) and complacency (m = 2.85); interestingly, the other three variables had very low means – financial strain (m = 2.38), perceived scarcity (m = 2.14), and restrictions (m = 1.76).

**Table 1:** Results of the descriptive analysis for the items to measure each construct

Construct/associated items	France		Iran	
**MV**	**SD**	**MV**	**SD**
**Vaccine acceptance**				
I want to be vaccinated against COVID-19	3.87	0.775	4.29	0.787
**Confidence**	**3.91**	**0.721**	**3.1**	**0.695**
I trust in effectiveness of COVID vaccines	2.98	0.664	3.27	0.754
I trust in safety of COVID vaccines	3.01	0.703	4.01	0.783
I trust in the system that delivers vaccines (health care workers, politics)	3.98	0.693	2.02	0.564
**Convenience**	**3.63**	**0.828**	**2.15**	**0.894**
Physical availability	4.01	0.910	1.98	0.868
Affordability	4.33	0.731	2.76	0.901
Atructural barriers	4.17	0.86	1.73	0.762
**Complacency**	**2.85**	**0.897**	**2.62**	**0.762**
Perception of COVID-19 risk	2.39	0.828	3.87	0.768
Perceived risks of vaccine	3.17	0.847	1.99	0.857
Vaccination not seen as necessary/importance	2.98	0.763	2.01	0.810
**Perceived scarcity**	**2.14**	**0.811**	**3.77**	**0.799**
There is a global shortage of COVID-19 vaccine	2.33	0.836	3.01	0.903
There is COVID-19 vaccine scarcity in my country	1.95	0.991	4.53	0.898
**Prohibition/restriction**	**1.76**	**0.858**	**4.32**	**0.901**
The government’s decision threatened my freedom regarding vaccination	1.47	0.814	4.02	0.978
This decision gives me a feeling of threatening my life	2.04	0.909	4.61	0.942
**Financial strain**	**2.38**	**0.937**	**3.87**	**0.836**
I lose my income during COVID-19 pandemic and lockdown (loss of income)	2.89	1.031	3.87	0.885
I lose my job insecurity during COVID-19 pandemic and lockdown (job insecurity)	2.17	0.896	3.56	0.921
My personal financial lifestyle is in risk (financial risk)	2.07	0.977	4.17	1.218

MV – mean value, SD – standard deviation

### Data analysis

The SmartPLS 3 software was used to perform PLS-SEM. PLS-SEM was selected primarily due to its ability to analyse the models including both formative and reflective constructs [54]. This method has become a useful technique in research because it enables to analyse complex models with formative and/or reflective constructs with non-normal data and small sample sizes [54].

We employed a combination of PLS-SEM analysis and multi-group analysis (MGA). We used PLS-SEM because it is more appropriate when conducting MGA [55,56]. In order to assess the conceptual model across two different contexts using PLS-SEM, this study evaluated the measurement model – by gauging the reliability and validity of reflective constructs – and the structural model – by evaluating R2, path coefficients, and the standardized root mean square residual (SRMR) values – as an approximate model fit for PLS-SEM [55]. Following our assessment of the measurement and structural models, we employed two different nonparametric methods, Henseler’s MGA [57] and the permutation test (58), for MGA. In advance of performing the MGA, we evaluated measurement invariance using measurement invariance for composite (MICOM), a newly developed approach to PLS-SEM. To the best of our knowledge, this empirical study constitutes the first on vaccination behaviour to apply these newly developed comparison techniques. In this way, this article makes a methodological contribution by employing advanced methods of analysis.

## RESULTS

The measurement model (outer model) assessment is presented in detail in the **Online Supplementary Document**, which makes it possible to claim that the model is a suitable way to asses our hypotheses.

### Structural model assessment

Prior to structural model assessment and the use of MGA to compare the path coefficients between the two groups, we established measurement invariance through a three-step MICOM approach, consisting of configural invariance, compositional invariance, and the equality of composite mean values and variances [57]. The MICOM results are shown in **Table 2**, revealing “a partial measurement invariance”, a requirement for comparing and interpreting the group-specific differences in PLS-SEM results revealed by the MGA [57].

**Table 2:** Results of measurement invariance testing

	Configural invariance (same algorithms for both groups)	Compositional invariance (correlation = 1)		Partial measurement invariance established	Equal mean assessment			Equal variance assessment			Full measurement invariance established
		**C = 1**	**CI**		**Differences**	**CI**	**Equal**	**Differences**	**CIi**	**Equal**	
CONF	Yes	1.000	(0.999-1.000)	Yes	0.047	(-0.179, 0.177)	Yes	0.324	(-0.228, 0.217)	No	No
CONV	Yes	0.0999	(0.997-1.000)	Yes	0.323	(-0.170, 0.171)	No	0.041	(-0.172, 0.176)	Yes	No
COMP	Yes	1.000	(1.000-1.000)	Yes	0.114	(-0.173, 0.175)	Yes	0.016	(-0.214, 0.197)	Yes	Yes
PSC	Yes	0.999	(0.999-1.000)	Yes	0.034	(-0.176, 0.175)	Yes	0.314	(-0.177, 0.181)	No	No
RES	Yes	1.000	(0.996-1.000)	Yes	0.124	(-0.175, 0.173)	Yes	0.146	(-0.202, 0.199)	Yes	Yes
FIN	Yes	1.000	(1.000-1.000)	Yes	0.157	(-0.176, 0.172)	Yes	0.056	(-0.174, 0.179)	Yes	Yes
VAC	Yes	1.000	(0.999-1.000)	Yes	0.375	(-0.175, 0.169)	No	-0.011	(-0.173, 0.174)	Yes	No

CONF – confidence, CONV – convivence, COMP – complacency, PSC – perceived scarcity, RES – restriction, FIN – financial strain, VAC – vaccine acceptance, C – correlation, CI – confidence interval

Once we validated our model, we examined model’s predictive accuracy and explanatory power by evaluating the values of R2 and Q2 for endogenous constructs for both groups. For French respondents, the R2 value was 0.634 and the Q2 value was 0.269, the values of 0.538 for R2 and 0.314 for Q2 respectively for Iranian respondents. According to behavioural research standards, a value of 0.2 for R2 is generally considered acceptable [54]. Therefore, for both groups, the results indicated acceptable in-sample predictive accuracy. **Table 3** shows the results of our structural model assessment and path relationships testing; the levels of statistical meaning of the coefficients were determined by a resampling and permutation procedure (with bootstrapping of 5000 samples and 5000 permutations). Results indicate that “restriction” and “perceived scarcity” have the most significant effects on Iranian vaccine acceptance but were insignificant among the French. Additionally, the results indicate that “confidence” and “financial strain” have positive and significant effects on COVID-19 vaccine willingness in both France and Iran. The findings also reveal that, while convenience has a significant and positive effect on French vaccine acceptance, it is insignificant among Iranians.

**Table 3:** Results of relationship testing

Relationships	Path coefficient		CI (95%), bias corrected		Path coefficient difference	*P*-value difference (one-tailed)		Supported
	**France**	**Iran**	**France**	**Iran**	**Henseler’s MGA**	**Permutation test**	
CONF→VAC	0.261*	0.217†	(0.042-0.157)	(0.168-0.313)	0.044	0.237	0.194	No
CONV→VAC	0.119†	-0.012	(0.187-0.468)	(-0.025,0.279)	0.131	0.035	0.021	Yes
COMP→VAC	0.097*	0.106*	(-0.237,0.163)	(-0.078,0.121)	-0.009	0.086	0.074	No
PSC→VAC	0.043	0.386*	(-0.021-0.203)	(0.188-0.463)	-0.343	0.001	0.005	Yes
FIN→VAC	0.052*	0.379†	(-0.019,0.216)	(0.034-0.262)	-0.327	1.000	0.002	Yes
RES→VAC	0.012	0.403*	(0.149-0.438)	(0.022-0.168)	0.391	0.981	0.018	Yes

CONF – confidence, CONV – convivence, COMP – complacency, PSC – perceived scarcity, RES – restriction, FIN – financial strain, VAC – vaccine acceptance, CI – confidence interval, MGA – multi-group analysis

*P < 0.001.

†*P* < 0.01.

**Table 3** displays the MGA results of two distinct nonparametric approaches: the permutation test [58] and Henseler’s bootstrap-based approach [57]. These two approaches to assessing variations in path coefficients between two groups are the most conservative PLS-SEM methods. Henseler’s MGA directly compares the group-specific bootstrap estimations from each bootstrap sample. Based on this approach, a *P*-value of differences between path coefficients under 0.05 or over 0.95 demonstrates significant differences at the 5% level between particular path coefficients in the two groups (ie, the French and Iranian people) [57,58]. The permutation test also returns a *P*-value; provided that the *P*-value is under 0.05, differences are significant at the 5% level. The results of our MGA, using both Henseler’s MGA and the permutation method, reveal significant differences between the French and Iranians with regard to the effects of convenience, perceived scarcity, financial strain, and restrictions/prohibition on vaccine acceptance. The results show these significant differences using both Henseler’s MGA and a permutation test. The effects of perceived scarcity, financial strain and restriction on vaccine acceptance are much higher for Iranian than for the French, while the effect of convenience on vaccine acceptance is far higher in France than in Iran.

In addition, for the two groups, we calculated the values of SRMR as an approximate model fit for PLS-SEM [57]. An SRMR value under 0.08 can be considered to be acceptable for PLS-SEM [57]. The results revealed SRMR model fit values of 0.072 and 0.067 for France and Iran, respectively.

## DISCUSSION

This study’s comparison between the effects of various influential factors on vaccine acceptance (France vs Iran) marks a unique theoretical contribution to the literature on vaccine acceptance. Its contribution is made more significant through the incorporation of three new constructs – perceived scarcity, prohibition of preferred vaccines, and financial strain (due to lack of state financial support during pandemic lockdowns) – all of which are common problems in lower-income countries. Additionally, this study addressed the paucity of research into COVID-19 vaccine acceptance in low-income countries. In fact, it was the first such study conducted on Iranian’s COVID-19 vaccine acceptance. In addition, this empirical study is one of the first to employ the MICOM approach in testing PLS-SEM measurement invariance and one of the first to use both Henseler’s MGA and Henseler’s permutation method to perform a multimethod MGA in vaccine acceptance studies. Thus, our results constitute a significant contribution to the existing literature on vaccine acceptance.

As mentioned earlier, MGA revealed significant differences in the effects of influential factors on vaccine acceptance across France and Iran. In other words, many determining factors are likely to be context-dependent. This finding could reflect health disparity across nations. These cross-cultural differences point to the importance of conducting additional research in this area that directly compares various effects across countries. Each country’s public health authorities may use these findings to the degree to which their country aligns with Iran or France. Based on the unique factors for vaccine-acceptance behaviours across the samples, national public health policy makers should aim to replicate our work to identify the influential factors of vaccine acceptance in their own contexts to enable more direct targeting.

Our results highlight the importance of confidence and complacency as determinants of vaccine acceptance in both France and Iran. Thus, attempts to combat misinformation, in particular that pertaining to vaccine safety, are necessary. Informational interventions, such as educational campaigns, can aid in addressing low confidence. Mass media is one the most critical sources of information about vaccines, and the rise in vaccine hesitancy stemming from safety misinformation coincides with the rise of social media. Additionally, earnest discussions with a trusted source can be effective. For most people, mainstream health care provides, nurses, therapists, and physicians constitute the most accepted source of vaccine information [59], meaning they can effectively impart a great deal of knowledge about the benefits and risks of the COVID-19 vaccines to public people, assure them about the safety and efficacy of vaccines, and increase vaccine confidence. Thus, health care professionals must have confidence in the safety, effectiveness, and necessity of vaccination.

In France, convenience has the second-highest effect on vaccine acceptance (β = 0.119), meaning that convenient vaccination programs and high-quality vaccination services would likely enhance vaccine acceptance in France and similar contexts. We expected the same result for Iranian vaccine acceptance. However, Iranians’ behaviours (eg, traveling to neighbouring countries for vaccination, waiting in long lines to get vaccinated) contradict previous findings suggesting that convenience is the most significant factor in vaccine adoption. Evidently, other variables overshadow vaccination behaviours in Iran. This study concluded that “perceived scarcity of vaccines” (β = 0.386), “prohibition of western vaccines/a lack of freedom in choosing their preferred vaccine” (β = 0.403), and “financial strain due to the lack of state support” (β = 0.379) impact Iranian vaccine acceptance. During pandemic lockdowns, Iranians felt more vulnerable than the French due to vaccine shortages and greater financial strain. Interestingly, they now seem more likely to accept the COVID-19 vaccine than the French, who did not need to struggle with vaccine shortages or a lack of state financial support. It is outside the scope of this study to assess the ethical dynamics surrounding the administration and distribution of scarce vaccines or the prohibition of critical public health products. However, our results may aid policymakers and social scientists in comprehending the psychological impacts of various vaccination policies and communication tactics and, in turn, assist them in controlling infectious illnesses like COVID-19 in the future. While mismanagement of the COVID-19 crisis by Iranian officials may have led to increased Iranian vaccine acceptance, we must not forget that politicizing public health issues and restricting access to approved vaccines will hinder the successful control of the current pandemic and will inevitably lead to public discontent. In spite of the willingness of the Iranian people to get vaccinated, the persistence of such policies would make reaching herd immunity an impossibility. Instead of politicizing a public health issue, the Iranian government must incorporate sensible diplomacy to remove every barrier in the way of vaccination.

Despite this study’s contributions, it suffers from some limitations that create interesting opportunities for future research. As with most samples collected online, our sample is likely restricted to people with a relatively high level of literacy. Thus, the generalizability of our findings to less-educated people is unclear. Similarly, our sample does not include individuals in institutionalized hospital care, prisons, or refugee centres or those who are difficult to reach (eg, offline people, homeless people). The exclusion of these members of society also limits the generalizability of our results. In addition, as mentioned before, many determining factors are likely to be context-dependent, so generalization of the results to other similar contexts should be addressed with added caveat and future studies should aim to replicate our work within and across alternative low and high-income contexts to identify the influential factors of vaccine acceptance in those contexts. Finally, this research was conducted over a short period of time; future research could use longitudinal data to allow for a deeper investigation into how, for example, the potential changes in vaccine acceptance in relate to “prohibition” and “scarcity”.

## Additional material:


Online Supplementary Document
