# Supplementary material for: Examining the effects of psychological reactance on COVID-19 vaccine acceptance: Comparison of two countries
Source: J Glob Health. 2022 Dec 21;12:05057. doi: 10.7189/jogh.12-05057 (PMC9767306; doi:10.7189/jogh.12-05057)
Supplement: Online Supplementary Document [file jogh-12-05057-s001.pdf]

ONLINE SUPPLEMENTARY DOCUMENT

Title: Examining the effects of psychological reactance on COVID-19 vaccine acceptance: A two Countries comparison

Authors: Fahimeh Hateftabar , Heidi J Larson, Vahideh Hateftabar

Supplement S1

Table S1. Descriptive Summary of Participants.

Characteristics	Frequency		Percentage (%)	
	France	Iran	France	Iran
Gender				
Male	346	417	52.1	58.4
Female	318	297	47.9	41.6
Age (Years)				
18-30	219	264	32.9	37
31-45	251	306	37.8	42.9
46-60	102	113	15.4	15.8
66 and above	92	31	13.9	4.3
Level of Education				
Lower than High School degree	87	83	13.1	11.6
High School degree	141	177	21.2	24.8
Undergraduate degree	235	251	35.4	35.2
Higher than Bachelor's degree	201	203	30.3	28.4
Employment status				
Full-time (self)/employed	320	246	48.2	34.5
Part-time (self)/employed	161	122	24.2	17.1
Retired	51	29	7.7	4.1
Unemployed	58	136	8.7	19
Student	50	65	7.5	9.1
Unemployed due to COVID- 19	24	116	3.6	16.2
History of chronic disease				
YES	73	87	11	12.2
NO	591	626	89	87.8
Experience of Severe COVID-19 in self or family				
YES	273	268	41.1	37.5
NO	391	446	58.9	62.5

Supplement S2

Measurement model assessment

Our evaluation of the measurement model entailed the assessment of the validity and reliability of the model's latent variables (LVs). It is important to note that there are two types of validity: convergent and discriminant. First, all reflective exogenous constructs were assessed, employing reliability and validity. We examined the composite reliability (CR), average variance extracted (AVE), and item loadings (λ) for each construct.

Generally, for indicator reliability to be regarded as acceptable, the loading should be greater than 0.7 (54). Those with a loading under 0.4 warrant deletion, while objects with a loading of 0.4–0.7 warrant deletion if they raise the CR and AVE above the threshold (54, 57). According to Table 1, most of the indicator loadings on their corresponding LVs for the respondents of the two groups exceeded 0.7. Several indicators loaded between 0.4 and 0.7, suggesting that they should be removed according to their AVE and CR. Additionally, we used the CR coefficient to evaluate the reliability of the construct; it must be over 0.7 to indicate reliability (54). According to Table 1, the CR for all of the LVs in the measurement model exceeded 0.7 for both groups. The findings show that the measurement model has acceptable reliability. To test the convergent validity of the measurement model for both groups with acceptable convergent validity, the AVE of the LVs should be more than 0.5 (54). According to Table 1, the constructs' AVEs exceeded 0.5, indicating that convergent validity was acceptable. Moreover, as the AVE and CR were higher than the threshold, we determined that the removal of indicators from the two foregoing models with loadings between 0.4 and 0.7 was unessential.

To evaluate discriminant validity, we used Fornell and Larcker's (1981) approach, which compares the square root of the AVE with the latent variable correlations. To achieve sufficient discriminant validity, all of the square roots of AVEs must be greater than the correlation coefficients of that construct with the other constructs (54). According to the inter-construct correlation matrix generated through PLS, all of the constructs demonstrate satisfactory discriminant validity (see Table 2). In addition, we employed the heterotrait-monotrait (HTMT) procedure to avoid problems of discriminant validity between the reflective constructs. In order to establish discriminant validity, the HTMT ratio must be below 0.9 (57). As shown in Table 3, for both study areas we have acceptable discriminant validity.

Table S2. Results of assessment of measurement model

Construct	Loading/weight		CR		AVE	
	France	Iran	France	Iran	France	Iran
COVID-19 vaccine acceptance (VAC)						
VAC1	0.906	0.956	0.914	0.927	0.720	0.632
Confidence (CONF)			0.887	0.901	0.759	0.592
CONF1	0.832	0.785				
CONF2	0.925	0.846				
CONF3	0.854	0.898				
Convenience (CONV)			0.946	0.920	0.642	0.625
CONV1	0.964	0.907				
CONV2	0.912	0.823				
CONV3	0.796	0.928				
Complacency (COMP)			0.950	0.951	0.785	0.649
COMP1	0.871	0.956				

COMP2	0.894	0.909				
COMP3	0.907	0.885				
Perceived Scarcity (PSC)			0.927	0.965	0.612	0.599
PSC1	0.825	0.914				
PSC2	0.762	0.886				
Restrictions (RES)			0.933	0.928	0.786	0.807
RES1	0.916	0.874				
RES2	0.796	0.885				
Financial strain (FIN)			0.896	0.915	0.709	0.771
FIN1	0.862	0.886				
FIN2	0.947	0.941				
FIN3	0.954	0.838				

Table S3. Discriminant validity: Fornell-Larcker criterion

	France							Iran						
Construct	CONF	CONV	COMP	PSC	RES	FIN	VAC	CONF	CONV	COMP	PSC	RES	FIN	VAC
CONF	0.814							0.823						
CONV	0.573	0.845						0.603	0.842					
COMP	0.589	0.571	0.862					0.497	0.688	0.832				
PSC	0.646	0.508	0.543	0.814				0.439	0.627	0.478	0.815			
RES	0.612	0.624	0.549	0.555	0.820			0.538	0.671	0.568	0.571	0.812		
FIN	0.603	0.688	0.554	0.606	0.644	0.863		0.621	0.713	0.553	0.527	0.636	0.872	
VAC	0.589	0.646	0.592	0.53	0.609	0.711	0.839	0.618	0.658	0.572	0.509	0.711	0.763	0.871

Table S4. Discriminant validity: $HTMT_{0.90}$.

Construct	France							Iran						
	CONF	CONV	COMP	PSC	RES	FIN	VAC	CONF	CONV	COMP	PSC	RES	FIN	VAC
CONF														
CONV	0.818							0.518						
COMP	0.434	0.721						0.502	0.711					
PSC	0.36	0.374	0.517					0.493	0.415	0.682				
RES	0.251	0.471	0.265	0.606				0.405	0.773	0.503	0.795			
FIN	0.310	0.545	0.512	0.126	0.601			0.611	0.554	0.461	0.528	0.608		
VAC	0.317	0.314	0.372	0.213	0.229	0.516		0.584	0.604	0.407	0.439	0.553	0.211	
